# Analyzing the defense response mechanism of *Atractylodes macrocephala* to *Fusarium oxysporum* through small RNA and degradome sequencing

**DOI:** 10.3389/fpls.2024.1415209

**Published:** 2024-07-22

**Authors:** Sen Fan, Yunjia Tang, Na Zhu, Qingling Meng, Yanguang Zhou, Yujin Zhao, Jingyan Xu, Chenxian Gu, Shijie Dai, Bo Zhu, Xiaofeng Yuan

**Affiliations:** ^1^ School of Life Sciences, Zhejiang Chinese Medical University, Hangzhou, China; ^2^ Future Health Laboratory, Innovation Center of Yangtze River Delta, Zhejiang University, Jiaxing, China; ^3^ School of Pharmaceutical Sciences, Zhejiang Chinese Medical University, Hangzhou, China

**Keywords:** *Atractylodes macrocephala*, *Fusarium oxysporum*, miRNA, target, high-throughput sequencing

## Abstract

**Introduction:**

*Fusarium oxysporum* is a significant soil-borne fungal pathogen that affects over 100 plant species, including crucial crops like tomatoes, bananas, cotton, cucumbers, and watermelons, leading to wilting, yellowing, growth inhibition, and ultimately plant death. The root rot disease of *A. macrocephala*, caused by *F. oxysporum*, is one of the most serious diseases in continuous cropping, which seriously affects its sustainable development.

**Methods:**

In this study, we explored the interaction between *A. macrocephala* and *F. oxysporum* through integrated small RNA (sRNA) and degradome sequencing to uncover the microRNA (miRNA)–mediated defense mechanisms.

**Results:**

We identified colonization of *F. oxysporum* in *A. macrocephala* roots on day 6. Nine sRNA samples were sequenced to examine the dynamic changes in miRNA expression in *A. macrocephala* infected by *F. oxysporum* at 0, 6, and 12 days after inoculation. Furthermore, we using degradome sequencing and quantitative real-time PCR (qRT-PCR), validated four miRNA/target regulatory units involved in *A. macrocephala–F. oxysporum* interactions.

**Discussion:**

This study provides new insights into the molecular mechanisms underlying *A. macrocephala*'s early defense against *F. oxysporum* infection, suggesting directions for enhancing resistance against this pathogen.

## 1 Introduction

In most eukaryotic organisms, small RNAs (sRNAs) are generated by RNase III-like enzymes such as dicer-like (DCL) proteins and incorporated into Argonaute (AGO) proteins to induce sequence-specific gene silencing (Huang et al., 2019). sRNAs are widely present in plants and their ability to regulate various biological processes including plant growth, development, and stress responses, which makes them increasingly valuable for managing plant diseases and pests (Si et al., 2020; Mekapogu et al., 2021). Plant sRNAs can be divided into two main categories, which are microRNA (miRNA) and small interfering RNA (siRNA).

miRNA, a class of non-coding sRNA molecules typically ranging from 20 to 24 nucleotides (nts) in length, plays a pivotal role in plant growth and development, response to environmental stress, and gene expression regulation. miRNA excels in its regulatory function by binding to complementary sequences on target mRNA molecules, leading to either mRNA degradation or translation inhibition (Jones-Rhoades et al., 2006). Accurate and definitive prediction of miRNA targets is crucial for comprehending miRNA responses. Degradome sequencing, known as Parallel Analysis of RNA Ends (PARE), is a high-throughput sequencing technique that identifies and validates the target mRNAs of miRNAs. This technique is indispensable for unraveling the involvement of miRNAs in development, disease pathogenesis, and response to environmental fluctuations (German et al., 2008). For instance, degradome analysis confirmed 195 mRNAs as targets for 194 miRNAs during radish root development, providing valuable insights into the intricate interplay between miRNAs and their targets (Liu et al., 2018).

The accumulating research underscored the critical role of miRNAs in bolstering plant defenses. In *Arabidopsis*, miR156 regulated plant aging by targeting the SQUAMOSA promoter binding protein-like (SPL) gene family (Wang et al., 2009). miR172 also played a key role in the morphogenesis of *Arabidopsis* flower organs by targeting transcription factors within the APETALA2 (AP2) gene family, promoting the formation of petals and stamens while inhibiting the formation of seals (Aukerman and Sakai, 2003). In *Gossypium*, miR477-CBP60A module played a key role in the late response to *verticillium* wilt, ghr-miR477 can directly cut GhCBP60A mRNA and regulate the expression of GhCBP60A at the post-transcriptional level (Hu et al., 2020). Additionally, in *Brassica*, miR1885 directly silenced the TIR-NBS-LRR class of resistance gene *BraTNL1* and mediated the silencing of *BraCP24* gene by targeting the Trans-Acting Silencing (TAS) gene *BraTIR1* for the production of trans-acting small interfering RNA (tasiRNA) (Cui et al., 2020).

*Atractylodes macrocephala*, known as Baizhu, is traditionally used as a herbal medicine in East Asia, especially China. It is valued for its restorative properties, often prescribed for addressing issues related to the gastrointestinal system, cancer, osteoporosis, obesity, and calming fetal movement (Zhu et al., 2018). However, *A*. *macrocephala* experiences continuous cropping obstacles, requiring a 5-10-year gap before the same land can be replanted. Among these, fungal diseases like root rot are the most severe issues associated with continuous cropping. The primary pathogen of this disease is *Fusarium oxysporum*, leading to the rotting of rhizomes and infecting other plants through the soil, causing the death of *A*. *macrocephala* (Peng Y. et al., 2021).

By combining sRNA sequencing and degradation sequencing, previous studies revealed the key regulatory network of miRNA response to salt stress in *Fraxinus velutina* (Liu et al., 2022), and identified miRNA and its target genes after wheat infection with *Fusarium graminearum* (Jin et al., 2020). These two sequencing technologies are helpful to better understand the mechanism of miRNA regulation in plants. In this study, we simultaneously performed miRNA sequencing and degradome sequencing to analyze the miRNAs and their targets involved in *F. oxysporum*–*A. acrocephala* interactions. Our results have provided useful data for further studies on the role of miRNAs in plant defense responses. This method can be used to study the effects of pathogen infection on host endogenous sRNAs, providing a theoretical basis for the prevention and treatment of *A*. *macrocephala* root rot disease.

## 2 Material and methods

### 2.1 Plant materials and inoculation

*A. macrocephala* seeds, sourced from traditional Chinese medicine research institute (independent institute) in Pan’an County, Zhejiang Province, underwent a pre-treatment of soaking for 12 hours, followed by spreading on moistened blotting paper and incubation at 25 ± 2°C for 15 days. Upon the emergence of the first leaf, seedlings were transferred to sterile soil and cultivated in a controlled environment chamber. Conditions were maintained at 25 ± 2°C, with a light intensity of 1600 lux, a 16/8 hour light/dark cycle, and 75% humidity, until the seedlings developed to the two-leaf stage.

*F. oxysporum* conidia were diluted to a concentration of 1×10^6^ cfu/mL in sterile water. *A. macrocephala* seedlings, at the two-leaf stage, were submerged in this spore suspension for 50 minutes before being replanted in sterile soil. Post-inoculation, at 0, 3, 6, 9, and 12-day intervals, the plants were extracted from the soil and washed with double-distilled water (ddH_2_O). Following this, the seedlings were disinfected for 3 minutes in 2% sodium hypochlorite (NaClO) (Yu et al., 2022) solution and subsequently rinsed thrice with ddH_2_O. Finally, the roots were excised and stored at -80°C for preservation.

### 2.2 Quantification of two standard curves for qRT-PCR

Whole genome DNA of *A. macrocephala* roots and *F. oxysporum* was extracted using FastPure Plant DNA Isolation Mini Kit (Vazyme, Nanjing, China) and Rapid Fungi Genomic DNA Isolation Kit (Sangon Biotech, Shanghai, China). The *Matk* gene of *A. macrocephala* and the *Prot* gene of *F. oxysporum* were used as internal reference genes for PCR amplification, using the primer pairs *Matk* F/R (10 μmol/L) and *Prot* F/R (10 μmol/L) (**Supplementary Table 1**). Concentrations of the amplified genes were quantified using the NanoDrop One™ (Thermo Fisher, MA, United States) and then adjusted to various concentrations for analysis by quantitative real-time PCR (qRT-PCR), with each experimental condition replicated thrice. Standard curves correlating absolute DNA content with cycle threshold were constructed for each gene.

### 2.3 Determination of *F. oxysporum* colonization

Total genomic DNA was isolated from the roots of *A. macrocephala* following inoculation with *F. oxysporum* at intervals of 0, 3, 6, 9, and 12 days, utilizing the FastPure Plant DNA Isolation Mini Kit (Vazyme, Nanjing, China). qRT-PCR analyses were conducted employing *Matk* F/R (10 μmol/L) and *Prot* F/R (10 μmol/L) primer pairs, with each experimental condition replicated thrice. The DNA content for both *A. macrocephala* and *F. oxysporum* was quantified using standard curves. The bar chart correlating the number of days post-inoculation (dpi) with the DNA content ratio of *F. oxysporum* to *A. macrocephala* within the inoculated tissue.

### 2.4 RNA isolation and small RNA sequencing

Total RNA was isolated from the roots of *A. macrocephala* employing TRIzol Reagent (Thermo Fisher, MA, United States) (Luz et al., 2016). The RNA concentration was quantified using the NanoDrop One™ (Thermo Fisher, MA, United States). Subsequently, approximately 2 µg of the total RNA served as the basis for constructing a sRNA library, utilizing the protocol outlined by the VAHTS Universal V6 RNA-seq Library Prep Kit (Vazyme, Nanjing, China), which was then sequenced on the Illumina NovaSeq 6000 platform (Modi et al., 2021).

### 2.5 Identification of known and novel microRNAs

Raw reads were processed using FastQC (http://www.bioinformatics.babraham.ac.uk) (Cortese et al., 2021) to eliminate adapters, junk sequences, repeats, low-complexity sequences, and prevalent non-coding RNA families. Clean reads were then annotated with sRNAs using Rfam 11.0 (http://rfam.org/) (Chen et al., 2009) and aligned against precursor and mature miRNA sequences from all plant species cataloged in miRBase 21.0 (http://www.miRbase.org/) (Ebhardt et al., 2010). The sequences mapped to the mature miRNA region were enumerated, followed by the prediction of their secondary structures. Subsequently, an expression level analysis of the known miRNAs for each sample was conducted, employing Transcript Per Million (TPM) for normalization of expression levels (Geng et al., 2014). Differential expression analysis of miRNAs across samples was carried out using DESeq (Zheng and Moriyama, 2013), which employs a negative binomial distribution model. Furthermore, expression pattern clustering of differentially expressed miRNAs (DEMs) was visualized using heatmaps. Finally, target gene prediction for differential miRNAs was performed using psRobot, resulting in the creation of a miRNA-target gene network diagram. All raw sequencing data were deposited into the NCBI Short Read Archive under the BioProject: PRJNA1123906 (Accession number: SRR29423013–SRR29423021).

### 2.6 Degradome library construction and target identification

Approximately 20 µg of total RNA was utilized to construct the degradome library (Zhai et al., 2020). The mRNA was isolated using magnetic beads and subsequently ligated to 3’ and 5’ adaptors. The mixture of biotinylated random primers and mRNA underwent reverse transcription, followed by PCR amplification. The resultant cDNA library was sequenced on an Illumina HiSeq 2000 platform (Bao et al., 2019). Raw sequencing data were analyzed with CleaveLand (Swetha et al., 2022) to identify potential cleavage targets. A degradation group density file was created by aligning sequence pairs with the cDNA database of the corresponding species. Splice site prediction software, GSTAr (Fan et al., 2015), was employed to infer the mRNA sequences of target genes that align with the sRNA sequences from the sequenced species. Finally, the target genes predicted from miRNAs were cross-referenced with mRNAs in the degradome density file to identify common mRNAs.

### 2.7 Gene ontology and KEGG analysis of target genes

Gene Ontology (GO) analysis was accomplished by https://www.bioinformatics.com.cn, an online platform for data analysis and visualization (Tang et al., 2023). Utilizing all genes as the background dataset, the software identified a candidate list of differentially expressed genes (DEGs) through Fisher’s exact test (Kofler et al., 2011). To mitigate the risk of false positives, *p*-values were adjusted employing four distinct multiple testing correction methods, including Bonferroni (Bano et al., 2024), Sidak (Sharma et al., 2022), and false discovery rate (FDR) (Kaler et al., 2019), setting a significance threshold of *p* < 0.05. Furthermore, Kyoto Encyclopedia of Genes and Genomes (KEGG) pathways analysis was also accomplished by this platform, pinpointing significant pathways impacted by DEGs.

### 2.8 Quantitative real-time PCR analysis

Inoculated and control roots were collected at 0, 6, and 12 dpi. First-strand cDNA synthesis was performed using the RevertAid First Strand cDNA Synthesis Kit (Thermo Fisher, MA, United States). In this study, qRT-PCR analyses were all conducted on the QuantStudioTM 3 (Thermo Fisher, MA, United States) utilizing SYBR Green I (Thermo Fisher, MA, United States) (Moradi et al., 2010). The PCR reaction volume was set at 20 µL. PCR conditions were as follows: 90°C for 5 min, 40 cycles (95°C for 10 s, and 60°C for 30 s), 94°C for 15 s, 60°C for 60 s and 95°C for 15 s. Fluorescence quantitative PCR data were normalized using the 2^-ΔΔCT^ method (Bubner and Baldwin, 2004), followed by statistical analysis with SPSS (Cheng et al., 2017). Group comparisons were conducted through one-way ANOVA, with results presented as the mean **±** standard error. Distinct letters signify statistically significant differences among treatment groups (*p* < 0.05). Primers targeting defense-related genes were designed using SnapGene (**Supplementary Table 1**). Each treatment was evaluated with three independent biological replicates.

## 3 Results

### 3.1 Colonization of *F. oxysporum* in the roots of *A. macrocephala*

**Figure 1:**
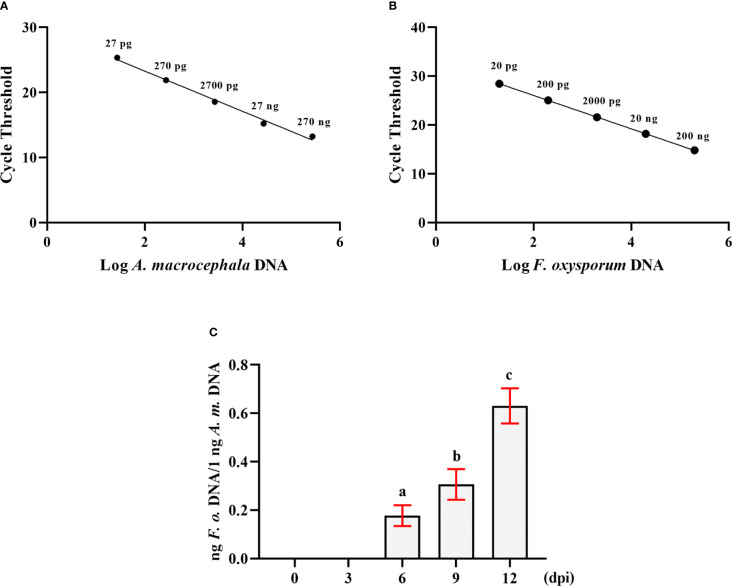
The double standard curve method was established to detect the amount of *F. oxysporum*. **(A)** Standard curve of the relationship between DNA content and CT value of *A. macrocephala*. **(B)** Standard curve of the relationship between DNA content and CT value of *F. oxysporum*. **(C)** qPCR analysis of the relative DNA content of *F. oxysporum* and *A. macrocephala*. Significant is the difference between “a”, “b” and “c” (*p* < 0.05).

DNA from *F. oxysporum* and *A. macrocephala* root tissues was extracted separately, and a range of primers was evaluated for their ability to amplify specific DNA sequences, ultimately selecting the *Matk* F/R and *Prot* F/R primer pairs for targeted amplification of *F. oxysporum* and *A. macrocephala* DNA, respectively. qRT-PCR utilized gradient-diluted DNA templates to construct standard curves, revealing a negative linear correlation between the CT value and the quantity of *A. macrocephala* DNA (R²=0.9909), as depicted by the equation Y = -3.087X+29.45 (**Figure 1A**). A similar negative linear correlation was observed for *F. oxysporum* DNA (R²=0.9997), represented by the equation Y = -3.408X+32 (**Figure 1B**). Utilizing the double standard curve method post-soaking treatment of *A. macrocephala* roots, colonization by *F. oxysporum* was detected on the 6th day, with the colonization level increasing over time (*p* < 0.05) (**Figure 1C**). This colonization timeline establishes a critical timeframe for the treatment of samples in subsequent experiments.

Following these findings, *A. macrocephala* root samples inoculated with *F. oxysporum* at 0, 6, and 12 dpi were selected for sRNA sequencing and degradation group sequencing.

### 3.2 Small RNA sequencing of *A. macrocephala*

**Figure 2:**
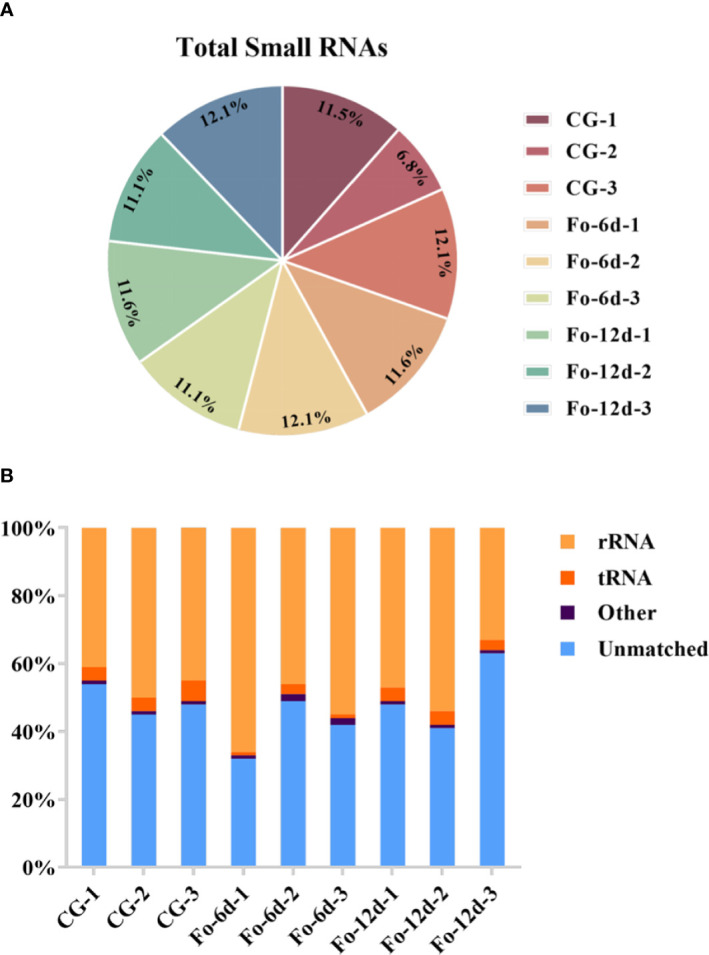
Profiling of sRNA sequencing in *A. macrocephala* plants infected by *F. oxysporum*. **(A)** Total clean reads distribution and radioactivity (% of total) in libraries at each time point between each experimental group and the control group. **(B)** Rfam classification table for sRNA.

To investigate the role of sRNAs in the response of *A. acrocephala* to *F. oxysporum* infection, we analyzed the dynamic changes in miRNA expression in *A. acrocephala* at 0, 6, and 12 days dpi utilizing the Illumina NovaSeq 6000. We constructed nine sRNA libraries, including CG (Control Group), Fo-6d (Inoculated with *F. oxysporum* for 6 days), and Fo-12d (Inoculated with *F. oxysporum* for 12 days), with three biological replicates for each treatment. The libraries yielded approximately 13.6 million reads each, ranging from 38,466,713 to 21,471,357 reads per library. After the removal of low-quality reads, we obtained unique reads ranging from 29,953,005 to 14,436,541 per library (**Figure 2A**; **Supplementary Table 2**). Concurrently, the Rfam classification of the overall sRNA was also acquired (**Figure 2B**).

**Figure 3:**
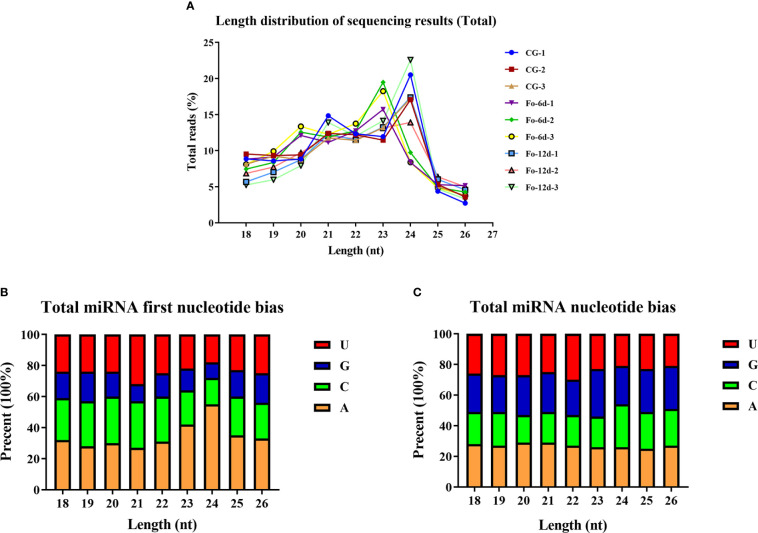
Total abundance of sRNA sequences in each size class and miRNA nucleotide bias analysis results. **(A)** Length distribution of sequencing results (total). **(B)** Results of total miRNA nucleotide bias. **(C)** Results of miRNA first nucleotide bias at each position.

Analysis of sRNA length distribution, ranging from 18 to 26 nts, indicated that 24 nt sRNAs were the most prevalent, followed by 23 nt sRNAs (**Figure 3A**). An analysis of nucleotide types revealed a general bias in the total average miRNA nucleotide composition (**Figure 3B**), with uracil (U) frequently occurring at the first position of 18-26 nt miRNAs (**Figure 3C**). Contrary to prevailing assumptions, the base composition analysis across different miRNA positions did not exhibit a discernible pattern, suggesting a deviation from the commonly held belief of inherent miRNA base bias.

### 3.3 Identification of novel and known microRNAs

**Figure 4:**
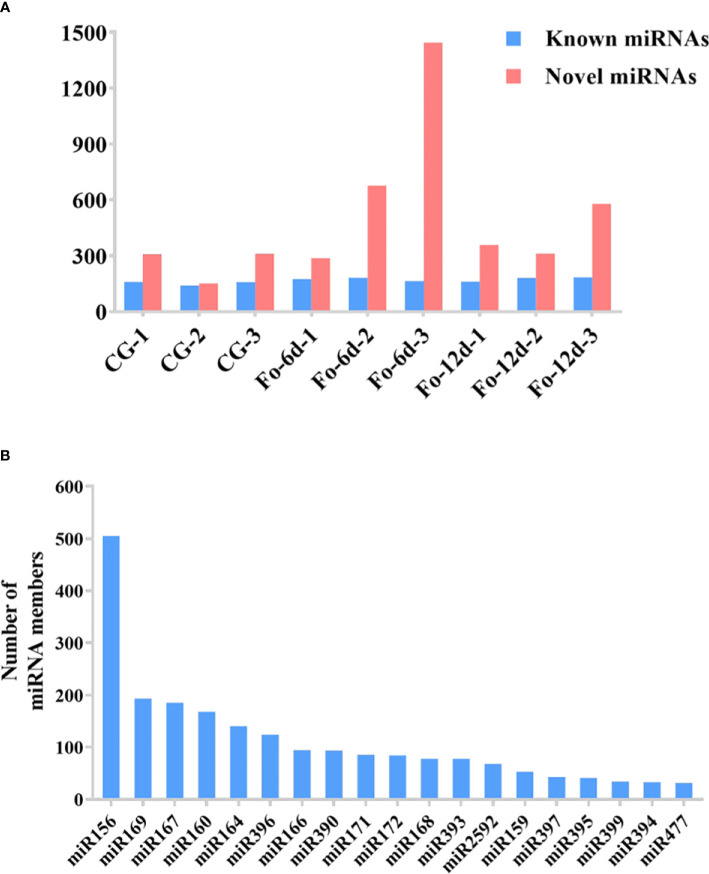
The statistics of miRNAs in the disparate sample. **(A)** Number of identified known and novel miRNAs. **(B)** Summary of the number of miRNA family members.

In the sRNA libraries, a total of 3,587 known miRNAs were identified through alignment with miRBase, as determined by the analysis of unique clean reads (**Figure 4A**). These miRNAs were categorized into 295 distinct families (**Supplementary Table 3**), with the miR156, miR169, miR167, and miR160 families each comprising over 150 members (**Figure 4B**).

**Figure 5:**
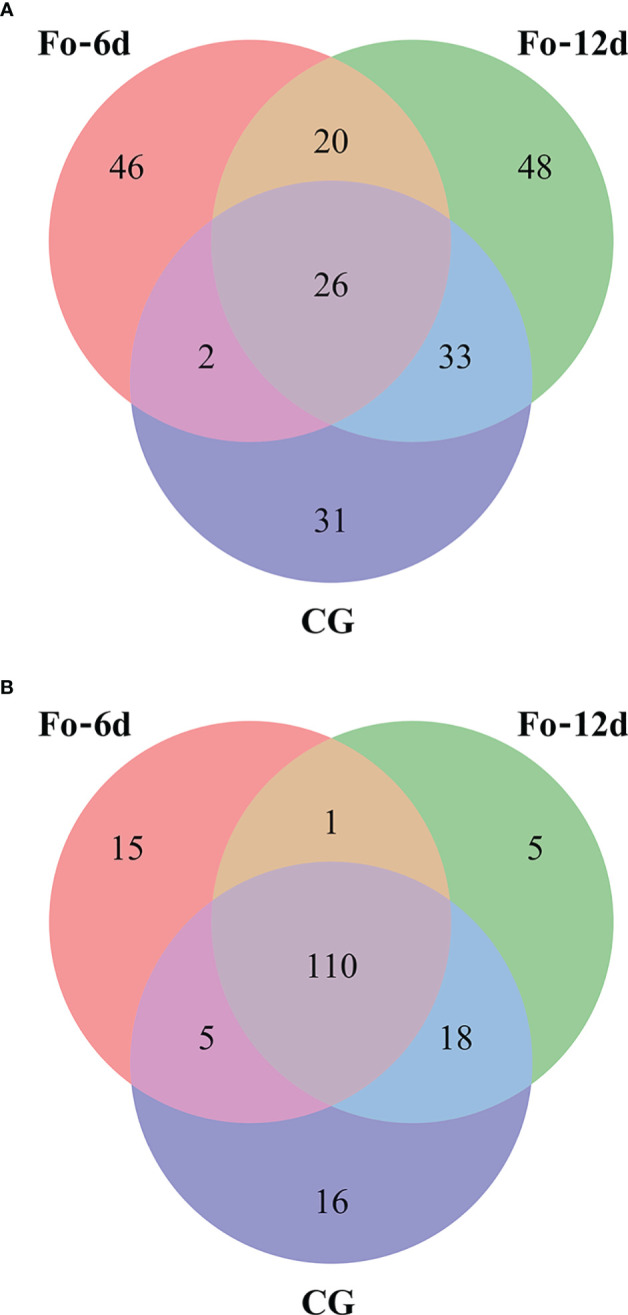
Venn-diagram analysis of the number and overlap of miRNAs in *A. macrocephala* plants infected by *F*. *oxysporum* at 0, 6, and 12 dpi. **(A)** Venn-diagram analysis of known miRNA **(B)** Venn-diagram analysis of novel miRNA.

Venn diagrams facilitated the comparison of miRNA sequences across the three study libraries (**Supplementary Table 4**). This comparison uncovered that the Fo-6d and Fo-12d groups contained 15 and 5 known unique miRNAs, respectively, whereas the CG harbored 16 miRNAs unique to it (**Figure 5A**). Moreover, an additional discovery revealed 46 and 48 novel unique miRNAs in the Fo-6d and Fo-12d groups, respectively, compared to 31 miRNAs found exclusively in the CG (**Figure 5B**).

### 3.4 Identification of differentially expressed miRNAs in *A. macrocephala* infected by *F. oxysporum*

**Figure 6:**
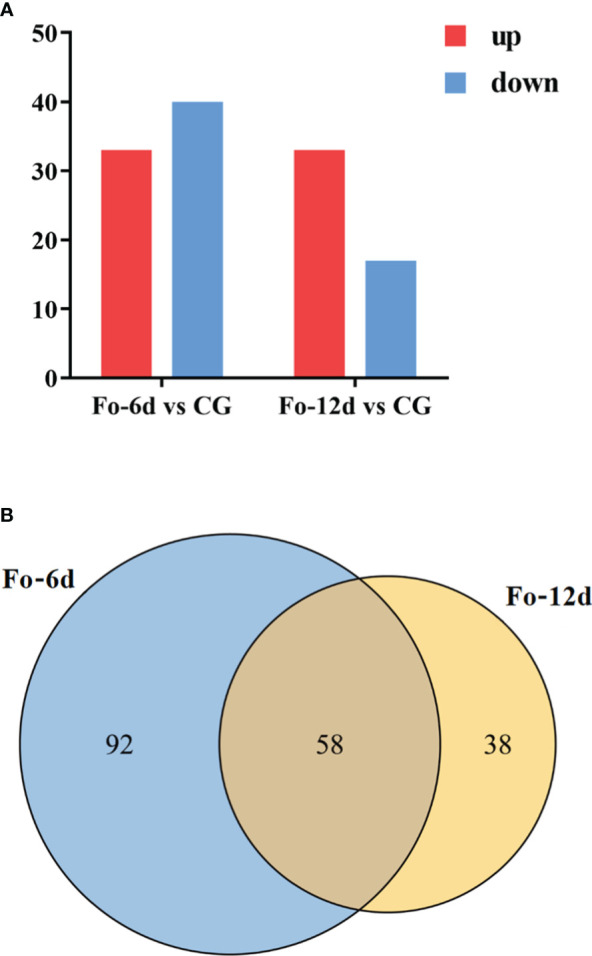
Statistics of differentially expressed miRNAs in different comparison groups. **(A)** Bar graph of differential miRNA in different comparison groups. **(B)** Venn-diagram of differential miRNAs in different comparison groups.

Following the identification of miRNAs, we performed a differential expression analysis to compare miRNA expression across the samples. DEMs identified at 6 dpi and 12 dpi, exhibited a log2 fold change exceeding 1.5 (*p <* 0.05). 73 DEMs including 33 upregulated and 40 downregulated miRNAs were detected at 6 dpi; 50 DEMs including 33 upregulated and 17 downregulated miRNAs were detected at 12 dpi (**Figure 6A**; **Supplementary Table 5**). The miRNA alterations were subtle, possibly due to the brief sample processing time impacting miRNA expression or the miRNAs’ efficient regulatory function triggering the defense response in *A. macrocephala.*

A Venn diagram illustrated the overlap of miRNAs between the different comparison groups (**Figure 6B**), showing that 58 miRNAs were common to both the Fo-6d and Fo-12d. Given the study’s 12-day treatment period and the minimal influence of factors like growth, development, and climate, these 58 miRNAs likely play a role specifically in the response to *F. oxysporum* infection.

To further explore the involvement of miRNAs in *A. macrocephala*’s defense mechanism, we analyzed DEMs consistently identified across both treatment groups. A heatmap of their expression patterns was created (**Supplementary Figure 1**), revealing the upregulation of most miRNAs in families miR156, miR396, and miR167 associated with the defense response. Conversely, miR477 family members exhibited transient early-stage downregulation (6 dpi), returning to baseline by 12 dpi. The pronounced variation in the newly identified differential DEMs was noteworthy. Nine DEMs, including Nov-m1089-3p and Nov-m0552-3p, were significantly downregulated, averaging a 5.5-fold change, while twelve, such as Nov-m1285-3p and Nov-m2005-3p, significantly upregulated, averaging a 5-fold change.

### 3.5 Target gene identification and function analysis of microRNAs by degradome sequencing

**Figure 7:**
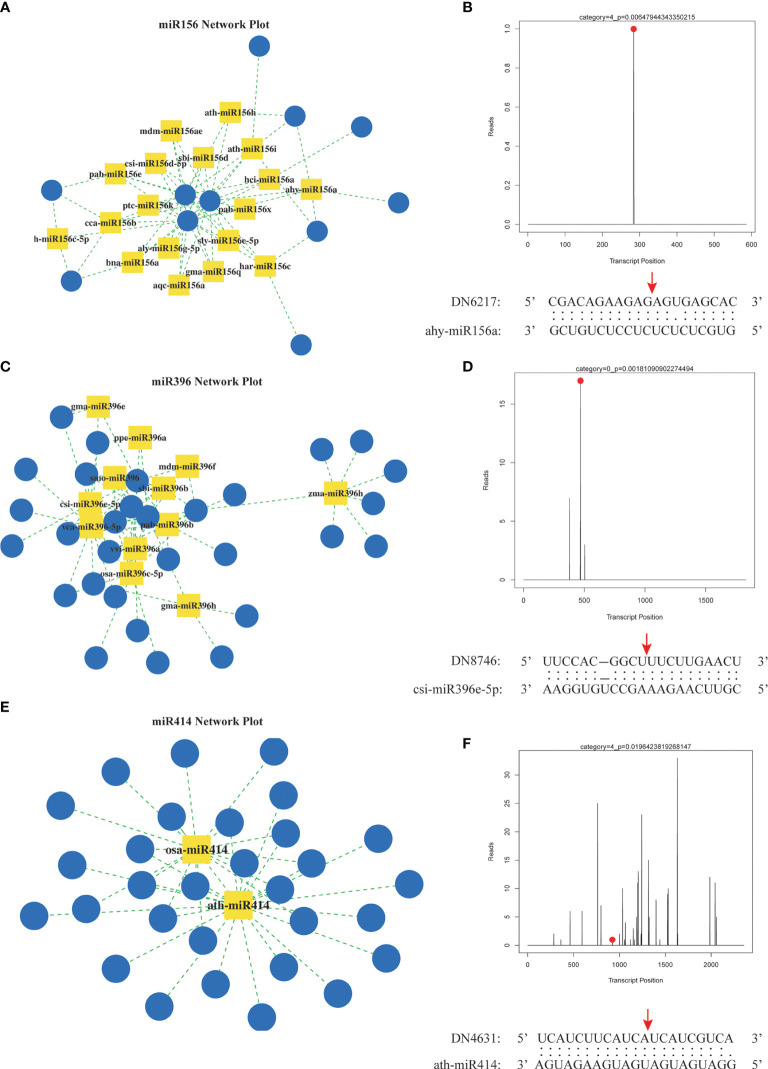
Prediction of miRNA target genes by degradation group sequencing. **(A)** miR156 regulatory network. **(B)** hy-miR156a cut DN6217 gene. **(C)** miR396 regulatory network. **(D)** csi-miR396-5p cut DN8746 gene. **(E)** miR414 regulatory network. **(F)** ath-miR414 cut DN4613 gene. Yellow square: miRNA, blue circle: mRNA, red dot and arrow: nucleotide cleavage site on target gene.

To elucidate the target genes of miRNAs in the *A. macrocephala*–*F. oxysporum* interaction, we created a mixed degradome library from the CG, Fo-6d, and Fo-12d. A remarkable 98.74% (19,220,589) of the raw reads from this library were successfully aligned with the genomic DNA, yielding 1,364,333 unique mappable reads. Of these, 5,704,143 (29.30%) unique reads were associated with transcripts of protein-coding genes in *A. macrocephala* (**Supplementary Table 6**). By leveraging degradome sequencing, we predicted miRNA target genes and developed a miRNA regulatory network diagram based on numerous miRNA–mRNA target pairs. This analysis highlighted three central miRNAs—miR156, miR396, and miR414—indicating their significant role in regulating a broad array of genes (**Figures 7A, C, E**, **Supplementary Table 7**). Additionally, degradome sequencing pinpointed the exact cleavage sites of miRNAs on their targets, identifying ahy-miR156a at the 284 bp position of DN6217, csi-miR396e-5p at the 469 bp position of DN8746, and ath-miR414 at the 924 bp position of DN4631 (**Figures 7B, D, F**).

**Figure 8:**
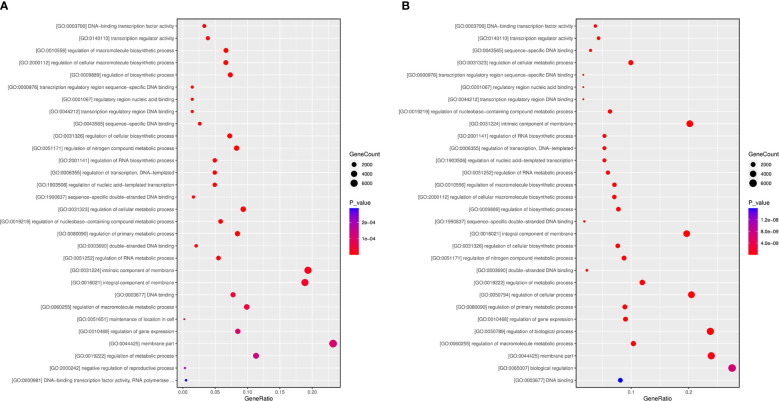
GO term enrichment at 6 **(A)** and 12 **(B)** dpi.

To further comprehend the regulatory impact of DEMs and their targets in the *A. macrocephala* and *F. oxysporum* interplay, we conducted GO enrichment analysis at 6 and 12 dpi. The analysis identified processes significantly enriched in plant defense, including the regulation of macromolecule biosynthetic process and regulation of cellular macromolecule biosynthesis. Particularly, the regulation of metabolic process emerged as a significant process at 6 dpi within the cellular component category, with the membrane part being the most enriched at 6 dpi (**Figure 8A**). At 12 dpi, in addition to the membrane part, biological regulation and regulation of biological processes were significantly enriched (**Figure 8B**). KEGG enrichment analysis further illuminated the involvement of DEGs in plant-fungus interactions, revealing that biosynthesis of secondary metabolites and ribosome pathways were notably enriched, supporting their role in plant defense at 6 and 12 dpi, respectively (**Supplementary Figure 2**; **Supplementary Table 8**).

### 3.6 Quantitative real-time PCR validation

**Figure 9:**
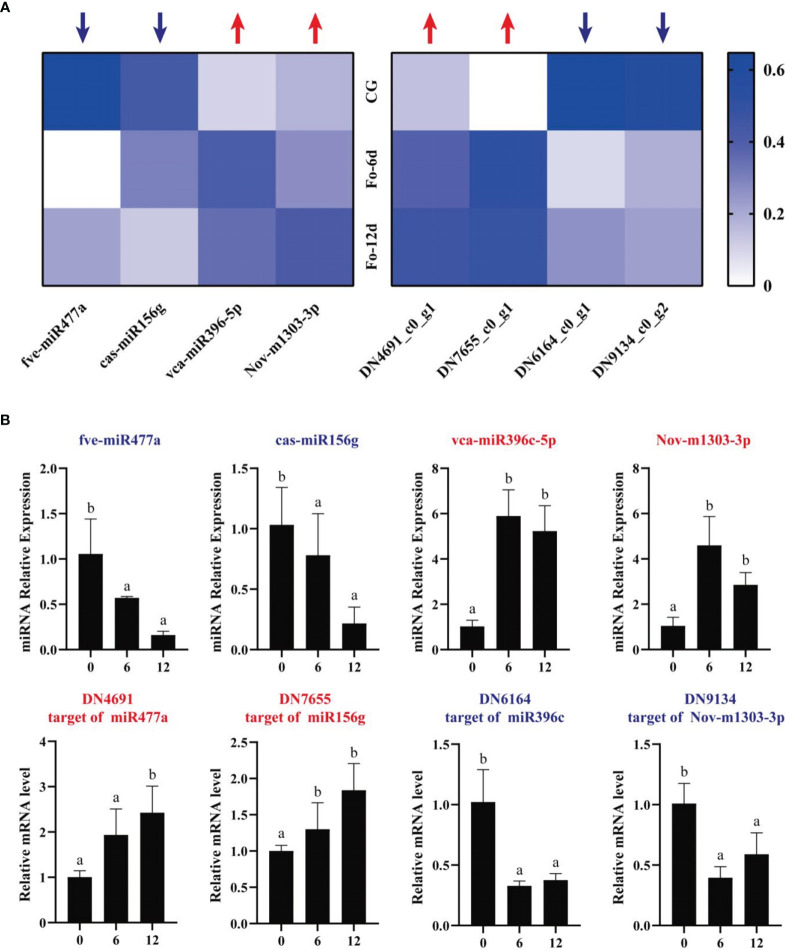
qRT-PCR analysis of the expression of miRNAs and their corresponding targets during *A. macrocephala*–*F. oxysporum* interaction. **(A)** Heatmap of expression levels for 4 pairs of mRNA–miRNA. **(B)** qRT-PCR detection of miRNA–mRNA target pair expression after *A. macrocephala* has been infected. Significant is the difference between “a” and “b” (*p* < 0.05).

To assess the miRNA/target regulatory dynamics, we analyzed the expression changes of miRNAs and their corresponding mRNAs during the *A. macrocephala*–*F. oxysporum* interaction via qRT-PCR. Four mRNA-miRNA pairs were selected for this analysis. The results showed a decrease in expression for fve-miR477a and cas-miR156g, while vca-miR396-5p and Nov-m1303-3p exhibited increased expression levels. Correspondingly, their target genes, DN4691_c0_g1 and DN7655_c0_g1, were upregulated, whereas DN6164_c0_g1 and DN9134_c0_g2 were downregulated, confirming a negative correlation between the miRNA-target gene pairs (**Figure 9A**). This pattern was consistent across qRT-PCR analyses of DEMs and their targets. Sequencing data analysis further supported that these four target genes were negatively influenced by their respective miRNAs (**Figure 9B**). Notably, vca-miR396-5p/DN6164_c0_g1 and Nov-m1303-3p/DN9134_c0_g2 exhibited significant regulatory activities at 6 dpi, whereas fve-miR477a/DN4691_c0_g1 and cas-miR156g/DN7655_c0_g1 showed more pronounced regulation at 12 dpi than at 6 dpi.

## 4 Discussion

*Fusarium oxysporum* is the main pathogenic fungus in *Atractylodes macrocephala* root rot disease. This fungus can also infect *Nicotiana tabacum*, causing a reduction in yield (Gai et al., 2021). Previous research established fluorescent quantitative PCR methods for detecting *F. oxysporum* in *Spinacia oleracea* (Okubara et al., 2013) and *Musa paradisiaca* (Zhang et al., 2013). By designing and selecting multiple primer pairs, we identified specific primers for amplifying *F. oxysporum* DNA and *A. macrocephala* DNA (*Matk* F/R and *Prot* F/R). Commonly, plasmids containing specific fragments are used to create standard curves for PCR quantification of fungal load, which can lead to biased results due to differences in sample volumes across groups. In this study, we utilized a hyperbolic method for relative quantification of fungal load to correct for volume biases, successfully detecting *F. oxysporum* colonization in the roots of *A. macrocephala* seedlings at 6 days (**Figure 1C**). This method can also be applied to the early prediction and control of root rot diseases in crops.

miRNAs are ubiquitous in plants and play crucial roles in regulating various biological processes, including growth, development, and stress responses (Si et al., 2020). Integrating transcriptome and sRNA sequencing methods provided insights into diseases such as *Cicer arietinum* wilt (Garg et al., 2019) and *Arabidopsis* wilt (Zhu et al., 2019). In this study, sRNA sequencing revealed a concentration of miRNA sequences at 24 and 21 nts (**Figure 3A**), similar to miRNA abundance in *Gossypium hirsutum* (Hu et al., 2020). Notably, the base composition analysis showed no apparent bias, which contrasts with previous reports of a first nucleotide bias towards uracil in miRNAs (Zhu et al., 2020) (**Figures 3B, C**). A total of 3,587 miRNAs were identified, distributed across 295 families, with the miR156, miR169, miR167, miR160, miR164, and miR396 families being the most abundant (**Figure 4B**), the number of miRNAs in the family may reflect the strength of the function of the family.

Integrating sRNA and degradome sequencing has significantly advanced our understanding of miRNA-regulated target genes in plant-pathogen interactions. This approach clarified miRNA regulatory networks in various systems, including *Brachypodium distachyon*–*Magnaporthe oryzae* (Peng W. et al., 2021), *Brassica napus*–*Sclerotinia sclerotiorum* (Jian et al., 2018), and *Gossypium hirsutum*–*Verticillium dahlia* (Zhang et al., 2015), providing insights into plant molecular improvements and pathogen control. In this study, we mapped the regulatory network of miRNA-target genes derived from degradome sequencing, with the miR156, miR396, and miR414 families serving as focal points (**Figures 7A, C, E**). miR156 and miR396 were notably abundant in sRNA sequencing and play crucial roles in plant activities. For example, the miR156-SPL module, by activating MdWRKY100, modulated plant salt stress tolerance (Ma et al., 2021). In *Arabidopsis*, reducing miR396 conferred broad resistance to fungal pathogens (Soto-Suárez et al., 2017). These findings suggest miR156 and miR396 play potential roles in *A. macrocephala*’s resistance to *F. oxysporum*. Additionally, the extensive gene targeting by miR414, enriched in pathways like ethylene signaling and polyketide metabolism, were reported for its high expression levels in several studies (Ma et al., 2018; Sobhani and Naghavi, 2018), underscoring the need for additional research into its function in *A. macrocephala*.

The incomplete nuclear genome data for *A. macrocephala* hinders research on gene and miRNA target functions. GO and KEGG pathway enrichment analyses of miRNA target genes have highlighted six key research areas, including ethylene-activated signaling pathway, auxin-activated signaling pathway, protein serine/threonine kinase activity, plant hormone brassinosteroid biosynthesis, alanine, aspartate and glutamate metabolism, and the plant MAPK signaling pathway. (**Supplementary 3A, B**). miR414 was notably enriched in ethylene signaling, making it a significant study focus. miR847 negatively regulated IAA28, boosting leaf and lateral root numbers via auxin signaling, closely related to root development in *A. macrocephala* (Wang and Guo, 2015). Brassinosteroid biosynthesis and the metabolism of alanine, aspartate, and glutamate suggested roles in active plant compound accumulation (Wang et al., 2021; Zhang et al., 2021), their relevance to the defense response of *A. macrocephal*a is yet to be clarified. The plant MAPK signaling pathway, essential for auxin-promoted lateral root formation (Zhu et al., 2019), indicated that understanding the miRNA-MAPK pathway could enhance resistance to *F. oxysporum*.

## 5 Conclusion

In this study, we utilized specific primers and the hyperbolic method to measure the relative fungal load of *F. oxysporum*, detecting colonization in the roots of *A. macrocephala* by day 6. Identified 3,587 known miRNAs in *A. macrocephala*, there were 73 and 50 DEMs at 6 and 12 dpi. miRNA families like miR156 and miR396 were involved in the expression and regulation of various physiological function proteins. GO and KEGG pathway enrichment analysis indicated that the target genes of differentially expressed miRNAs are enriched in spliceosome and plant-pathogen interaction pathways, suggesting that splicing mechanisms and pathogenesis-related proteins play important roles in the defense response of *A. macrocephala*. Additional qRT-PCR studies on four miRNAs and their potential target genes revealed the important roles of splicing mechanisms and disease-related proteins in the plant’s defense response, providing new insights into the molecular mechanisms of *A. macrocephala*’s early defense against *F. oxysporum* infection.

## Data availability statement

The datasets presented in this study can be found in online repositories. The names of the repository/repositories and accession number(s) can be found below: https://www.ncbi.nlm.nih.gov/, BioProject PRJNA1123906, SRR29423013 - SRR29423021.

## Author contributions

SF: Writing – original draft, Writing – review & editing, Conceptualization, Formal analysis, Investigation, Methodology, Validation. YT: Writing – original draft, Writing – review & editing, Conceptualization, Formal analysis, Investigation, Methodology, Validation. NZ: Formal analysis, Investigation, Writing – review & editing. QM: Formal analysis, Investigation, Writing – review & editing. YaZ: Formal analysis, Investigation, Writing – review & editing. YuZ: Formal analysis, Investigation, Writing – review & editing. JX: Formal analysis, Investigation, Writing – review & editing. CG: Validation, Writing – review & editing. SD: Validation, Writing – review & editing. BZ: Writing – review & editing. XY: Writing – review & editing.
