# Supplementary figures and images for: Analyzing the defense response mechanism of Atractylodes macrocephala to Fusarium oxysporum through small RNA and degradome sequencing
Source: Front Plant Sci. 2024 Jul 22;15:1415209. doi: 10.3389/fpls.2024.1415209 (PMC11298489; doi:10.3389/fpls.2024.1415209)

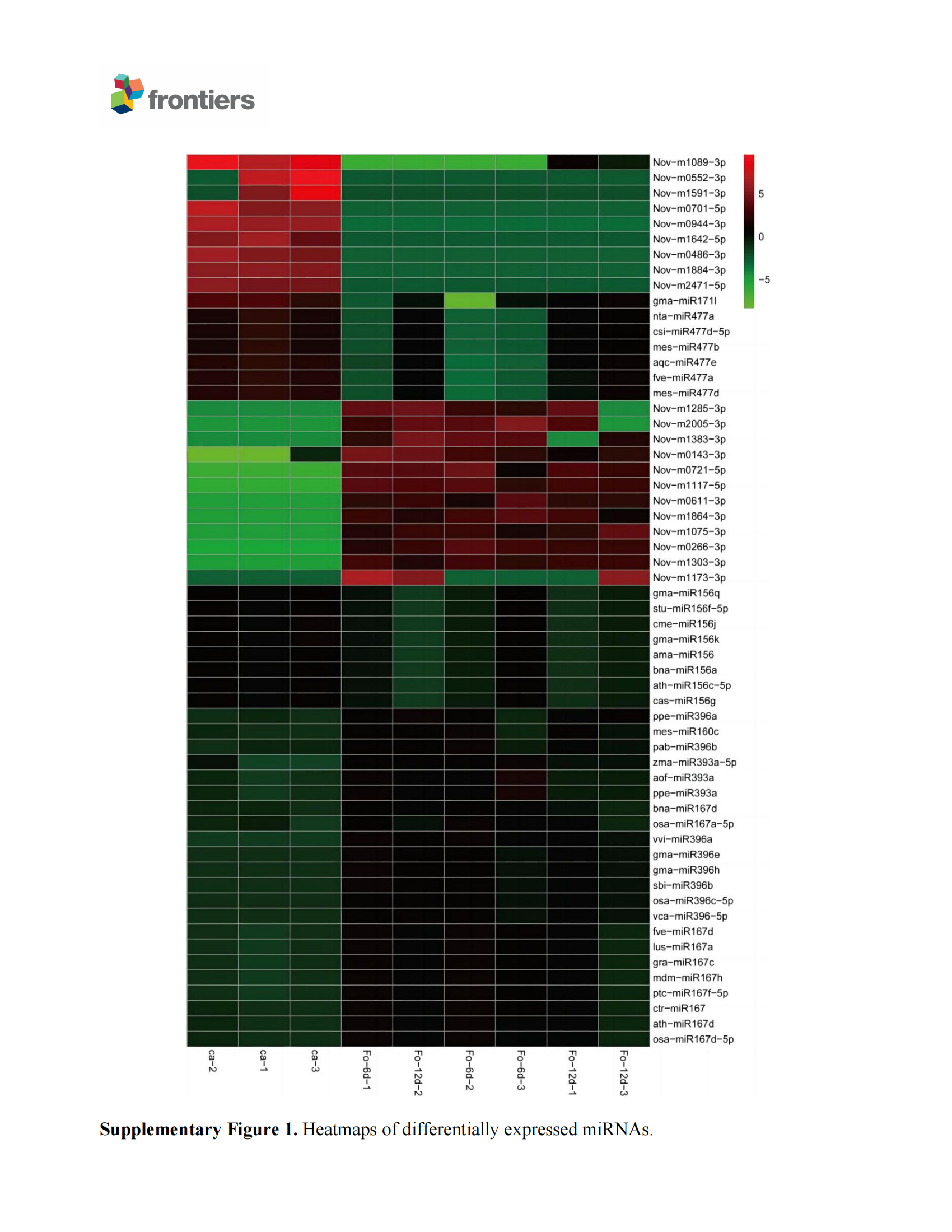

Supplement: Supplementary file 1 [file Image_1.tif]

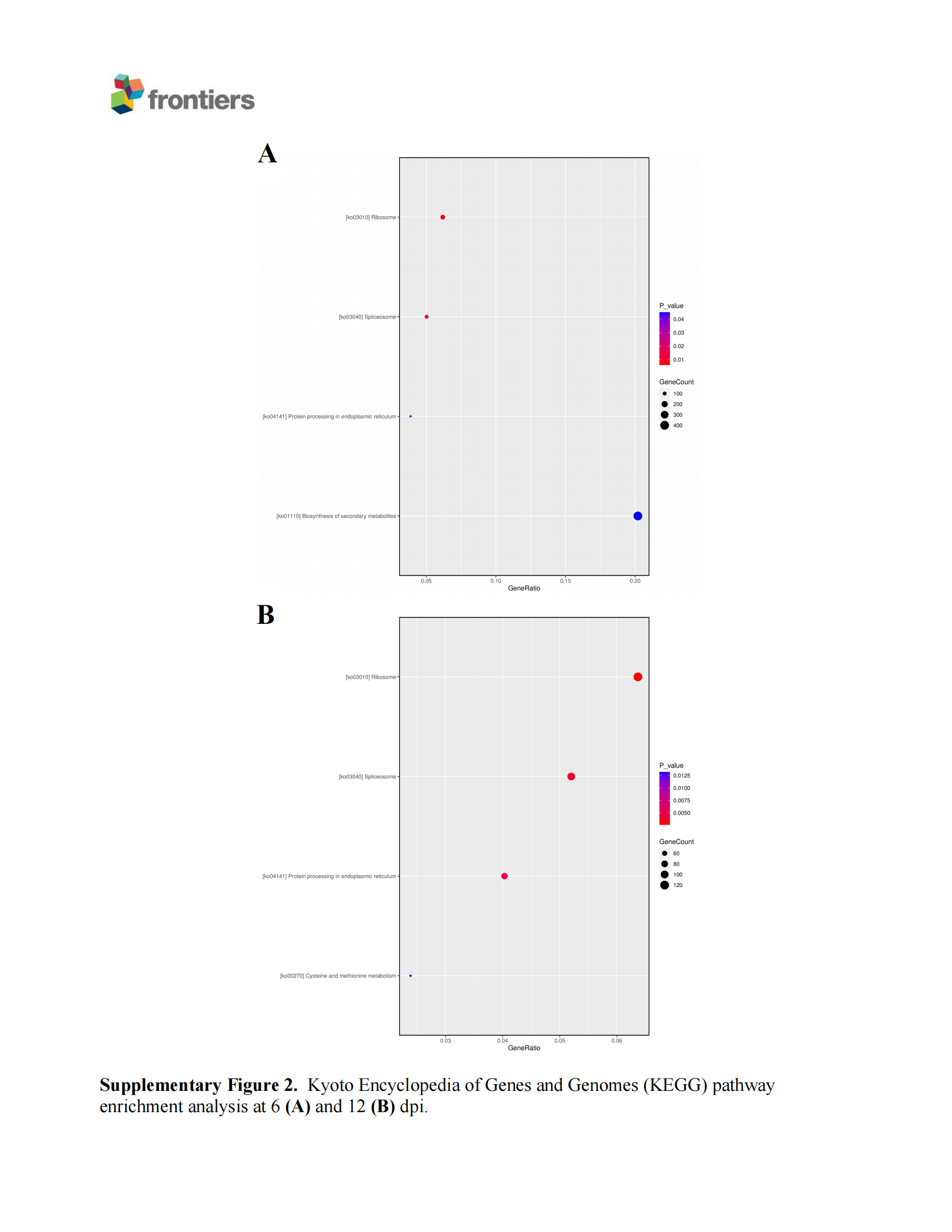

Supplement: Supplementary file 2 [file Image_2.tif]

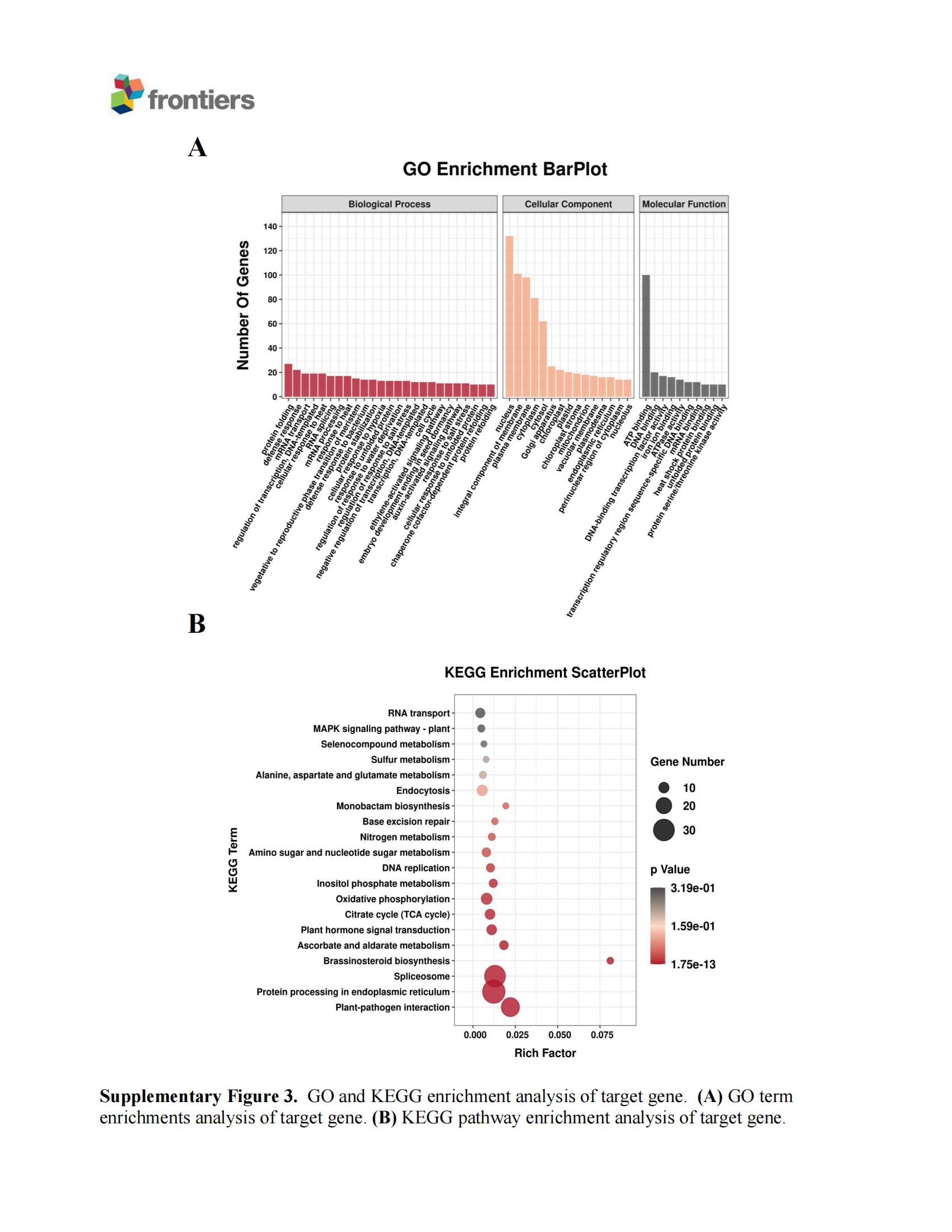

Supplement: Supplementary file 3 [file Image_3.tif]
